# Multiple aneurysms in subarachnoid hemorrhage - identification of the ruptured aneurysm, when the bleeding pattern is not self-explanatory - development of a novel prediction score

**DOI:** 10.1186/s12883-020-01655-x

**Published:** 2020-02-29

**Authors:** Alexis Hadjiathanasiou, Patrick Schuss, Simon Brandecker, Thomas Welchowski, Matthias Schmid, Hartmut Vatter, Erdem Güresir

**Affiliations:** 1 Department of Neurosurgery, Rheinische Friedrich-Wilhelms-University, Sigmund-Freud-Str. 25, 53127 Bonn, Germany; 2 Institute for Medical Biometry, Informatics and Epidemiology, Faculty of Medicine, Rheinische Friedrich-Wilhelms-University, Bonn, Germany

**Keywords:** Multiple intracranial aneurysms, Rupture prediction, Subarachnoid hemorrhage

## Abstract

**Background:**

In aneurysmal subarachnoid hemorrhage (SAH) and multiple intracranial aneurysms (MIAs) identification of the bleeding source cannot always be assessed according to the hemorrhage pattern. Therefore, we developed a statistical model for the prediction of the ruptured aneurysm in patients with SAH and multiple potential bleeding sources at the time of ictus.

**Methods:**

Between 2012 and 2015, 252 patients harboring 619 aneurysms were admitted to the authors’ institution. Patients were followed prospectively. Aneurysm and patient characteristics, as well as radiological findings were entered into a computerized database. Gradient boosting techniques were used to derive the statistical model for the prediction of the ruptured aneurysm. Based on the statistical prediction model, a scoring system was produced for the use in the clinical setting. The aneurysm with the highest score poses the highest possibility of being the bleeding source. The prediction score was then prospectively applied to 34 patients suffering from SAH and harboring MIAs.

**Results:**

According to the statistical prediction model the main factors affecting the rupture in patients harboring multiple aneurysms were: 1) aneurysm size, 2) aneurysm location and 3) aneurysm shape. The prediction score identified correctly the ruptured aneurysm in all the patients that were used in the prospective validation. Even in the five most debatable and challenging cases assessed in the period of prospective validation, for which the score was designed for, the ruptured aneurysm was predicted correctly.

**Conclusions:**

This new and simple prediction score might provide additional support for neurovascular teams for treatment decision in SAH patients harboring multiple aneurysms. In a small prospective sample, the prediction score performed with high accuracy but larger cohorts for external validation are warranted.

## Background

Up to 20% of patients suffering from aneurysmal subarachnoid hemorrhage (SAH) are harboring multiple intracranial aneurysms (MIAs) [1, 2]. The bleeding source can be assessed by cranial imaging findings, e.g. according to the pattern of the hemorrhage in computed tomography (CT) in most cases. Nehls et al. described an algorithm in 1985 helping to identify the ruptured aneurysm according to mainly imaging findings [3]. However, according to this algorithm the ruptured aneurysm was identified with the highest accuracy when focal accumulation of blood in CT was seen in proximity to the ruptured aneurysm. While in such cases treatment decision of the ruptured aneurysm is unambiguous, in some cases with no definite hemorrhage pattern the identification of the ruptured aneurysm can be challenging, for example, when the hemorrhage pattern has no lateralization and two or more aneurysms are in immediate proximity. As a result of this issue, we developed a statistical model for the prediction of the ruptured aneurysm, which could provide support in daily clinical routine for neurovascular teams.

## Methods

### Study population

Between 2012 and 2015, 252 patients harboring 619 aneurysms were admitted to the authors’ institution. SAH was diagnosed by CT or lumbar puncture. CT-angiography (CT-A) and additional digital subtraction angiography (DSA) were performed in order to identify the bleeding source. Information, including patient characteristics on admission and during treatment course, treatment modality, aneurysm size, shape, location and further radiological features, were collected prospectively and entered into a computerized database. Exclusion criteria were (1) insufficient quality of CT-A/DSA to evaluate aneurysm shape or size, and (2) the presence of fusiform, mycotic or partially thrombosed aneurysms. Follow up was standardized for all patients with clinical follow ups at 6, 12 months and then annually. At 6 months the first follow up was combined with DSA and MRI (magnetic resonance imaging), which was then repeated annually. All aspects of this study were approved by the local ethics committee and because of the character of the study, patient consent was not required.

### Identification of the ruptured aneurysm

The ruptured aneurysm was treated in all patients with SAH. The neurovascular team at the authors’ institution identified the aneurysm with the highest rupture probability according to the CT findings (i.e. pattern of hemorrhage, intracerebral hemorrhage (ICH)) and aneurysm characteristics (shape, size, location) on patient admission. In order to standardize the definition of aneurysm shape, all multilobulated aneurysms or aneurysms with a bleb were considered as irregularly shaped; all others were considered as regularly shaped. In uncertain cases, the aneurysm shape was decided in an interdisciplinary meeting of the neurovascular team.

According to the findings, treatment of the presumably ruptured aneurysm was performed.

After treatment, validation of the prediction score was performed in two ways: surgically treated aneurysms were directly inspected, and the bleeding source was identified. In endovascularly treated aneurysms the identification of the ruptured aneurysm in the prospective cohort was made according to the bleeding pattern or further CT findings, e.g. ICH and long-term follow-up. The bleeding source, i.e. the ruptured aneurysm, was unambiguous in all aneurysms treated endovascularly.

### Evaluation of the prospective cohort after development of the score

Independent of the decision of the neurovascular team, and independent to the aneurysm treatment, information of all aneurysms (aneurysm size, location and shape) included in the prospective validation were transferred from the neuroradiologist to an author (AH) in written form for the calculation of the prediction score.

### Data

Aneurysm location was divided into five regions in order to simplify the scoring system: anterior cerebral artery (AA) including anterior communicating artery (AcomA), internal carotid artery excluding posterior communicating artery (ICA), posterior communicating artery (PcomA), middle cerebral artery (MCA), and the posterior circulation. The maximum aneurysm diameter was defined as aneurysm size. Furthermore, aneurysm shape was categorized in regular and irregular shape. Irregular shape was defined if the aneurysm was lobulated or a bleb was found. All other cases were defined as regular shaped aneurysms.

For statistical analysis each aneurysm was treated as a separate observation, implying that each of the patients could contribute several observations (= aneurysms) to the analysis data set. The outcome variable for each observation was binary (rupture / no rupture). Some of the predictor variables, namely size, shape and location, varied across the aneurysms. Additionally, patient-specific predictor variables were considered, e.g., age, smoking status, hypertension (AHT), number of aneurysms per patient and number of additional aneurysms per region. Only one variable (namely the shape of aneurysm) contained missing values. The number of missing values in this variable was 23 (2.91%). Due to this very low number, and since all other variables were completely observed, we did not apply multiple imputation but choose the more straightforward approach of imputing missing aneurysm shapes by single logistic regression conditional on all other variables (imputed values: 18 regular and five irregular). After derivation of the score, the score was prospectively validated in a consecutive cohort of patients with SAH and MIAs.

### Statistical analysis

A component-wise gradient boosting algorithm with linear base learners [4] was used to derive the prediction score. This modeling approach simultaneously selects variables that are relevant for prediction and estimates the relationship between the outcome and the predictor variables. A binomial distribution with logistic link function was used to account for the binary structure of the outcome. Of note, the statistical model underlying the gradient boosting approach is defined in terms of a score that has exactly the same structure and interpretation as the linear score of a classical logistic regression model. Compared to the latter approach, the main difference (and advantage) of the gradient boosting approach is the algorithmic procedure that is used to fit the logistic model (i.e., to estimate its coefficients). In particular, gradient boosting contains the aforementioned built-in procedure for data-driven variable selection. We note that major overfitting issues due to variable selection are unlikely in this case, as the number of patients (*n* = 252) is sufficiently large compared to the number of candidate variables (10, i.e. 25 patients per variable). In addition, to counteract the variability induced by variable selection, the coefficient estimates obtained from gradient boosting are regularized such that they automatically improve predictive performance and reduce overfitting. The coefficients of logistic regression are included in the search space as a special case of the more flexible gradient boosting model. By definition, the prediction function of our model included an aneurysm-specific part (“prediction score”), which was given by a linear combination of the variables size, shape and location of the aneurysm. It also included a patient-specific part that was given by a linear combination of age, smoking status, AHT, number of aneurysms per patient and number of additional aneurysms per region. To account for the fact that some patients had multiple aneurysms whose measurements may be dependent, a patient-specific random effect was included in the boosting model [5].

For prediction, the boosting model was used to compute the rupture probabilities for all aneurysms. These predicted probabilities included all variables selected for the model, i.e., aneurysm-specific and patient-specific variables. For each patient the aneurysm with maximum probability was predicted as the one to rupture. Note that the probabilities of rupture directly correspond to the magnitude of the aforementioned aneurysm-specific part of the model’s prediction function. On the patient level (where patient-specific variables such as age are constant), a simplified strategy is therefore to predict the aneurysm with maximum value of the aneurysm-specific prediction score as the one to rupture.

**Fig. 1:**
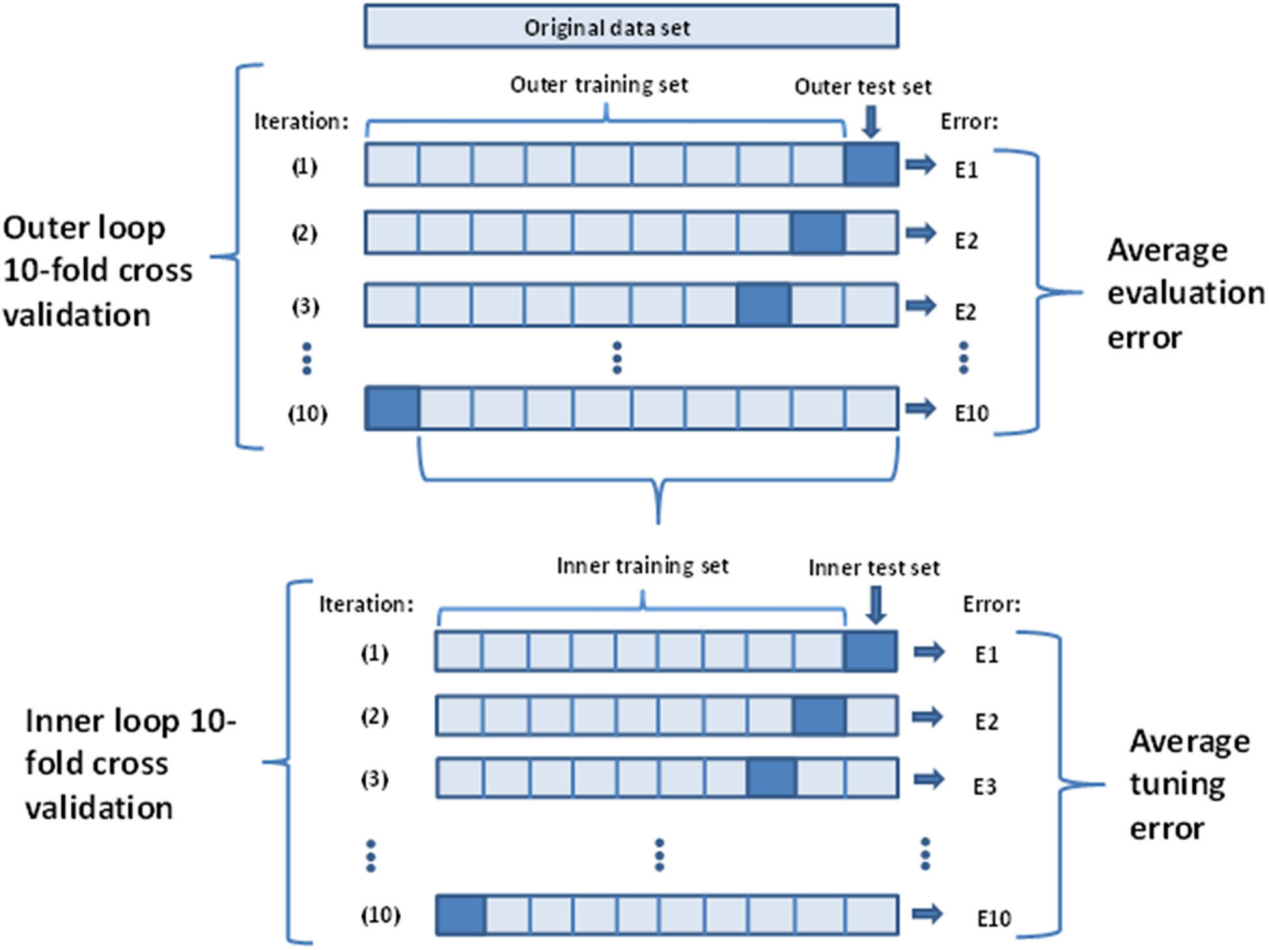
Ten-fold cross-validation

To evaluate the prediction accuracy of the proposed scoring system, ten-fold cross-validation was carried out (Fig. 1) [6]. To this purpose, the data were subdivided into ten mutually exclusive subsets (“folds”) of equal size. Patients in each fold were used to evaluate a boosting model fitted to the union of the respective other nine folds (“learning samples”). Predictions were evaluated on the patient level by (i) selecting the aneurysm that was predicted to rupture according to the value of its aneurysm - specific prediction score, as described above, and (ii) by evaluating whether the selected aneurysm coincided with the true ruptured aneurysm. Prediction accuracy was summarized by considering the percentages of correctly classified ruptures on the patient level. In addition, the area under the receiver operating characteristic curve (AUC) was calculated. The number of boosting iterations, which is the main tuning parameter of gradient boosting, was determined using additional ten-fold cross-validation within each of the learning samples.

The final prediction score was obtained by fitting a gradient boosting model to the complete data set, using ten-fold cross validation on the complete data to select the optimal number of boosting iterations.

## Results

### Summary of the retrospective data

Overall, data of 252 patients harboring 619 aneurysms were analyzed. Detailed data on patient and aneurysm characteristics is shown in Table 1.

**Table 1:** Patient and aneurysm characteristics

		MIAs (%)
Pat. No.		252
Aneurysm No.		619
Sex (female/male)		195/57 (46/13)
Median age (y) ± SD		53 ± 12,8
AHT (%)		112 (44%)
Smoker (%)		89 (35%)
Aneurysm size (mm)		6 ± 5
Aneurysm shape	**Regular (%)**	512 (83)
	**irregular (%)**	107 (17)
Aneurysm localization (%)		
AcomA + AA		119 (19)
PcomA		85 (14)
Posterior circulation		72 (12)
MCA		234 (38)
ICA w/o PcomA		109 (17)
Aneurysms frequency		
2		174 (41%)
3		49 (11%)
4		22 (6%)
5		6 (1%)
6		1 (1%)

*AHT* arterial hypertension, *AcomA* anterior communicating artery, *AA* anterior cerebral artery, *ICA* internal carotid artery, *MIAs* multiple intracranial aneurysms, *MCA* middle cerebral artery, *PcomA* posterior communicating artery, *SAH* subarachnoid hemorrhage, *SD* standard deviation, *SIAs* single intracranial aneurysms

### Statistical prediction model

**Fig. 2:**
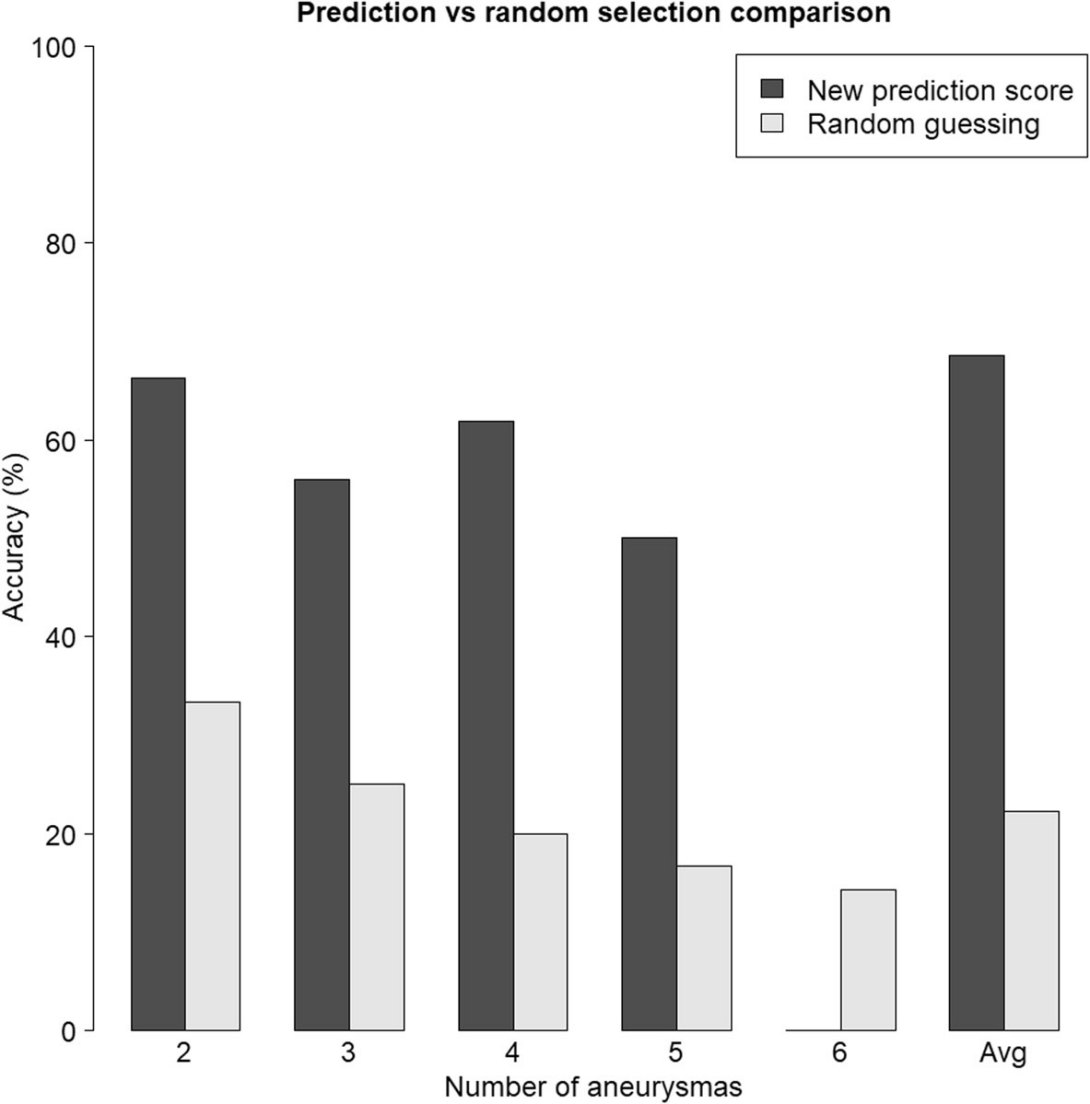
Percentages of correctly classified ruptures obtained from ten-fold cross-validation and probabilities of identifying the true ruptured aneurysms by random guessing

Figure 2 presents the percentages of correctly classified ruptures obtained from ten-fold cross-validation. Results are stratified according to patient groups having two, three etc. aneurysms. Furthermore, Fig. 2 contains the probabilities of identifying the true ruptured aneurysms by random guessing. It is seen that the correct classification rates obtained from the scoring system are substantially higher on average than the respective rates that would be obtained from random guessing. The probability of correct findings declined as the number of aneurysms increased. The only patient subgroup where the scoring system performed worse than random guessing is the group with six aneurysms. There was, however, only one patient with six aneurysms in the sample, so that the correct classification rate in this subgroup could either take the values 0% or 100%. The average AUC values were 80.89% on the test data (accuracy 68.58%), 81.94% on the training data (accuracy 68,37%) and 82.07% on the complete data (accuracy 68,63%), suggesting very little indication of overfitting. The final aneurysm - specific prediction score obtained from fitting a gradient boosting model to the complete data set was given by.

Aneurysm - specific prediction score = A + B + C, where.

A = 0.0427 x size of aneurysm (mm).

B = 0 if Location = AcomA and AA.

or − 0.0104 if location = PcomA.

or − 0.1831 if location = posterior circulation.

or − 0.4055 if location = MCA.

or − 0.5973 if location = ICA without PcomA.

C = 0 if shape = regular.

or 0.5387 if shape = irregular.

By definition, higher scores represent a higher risk of rupture.

**Fig. 3:**
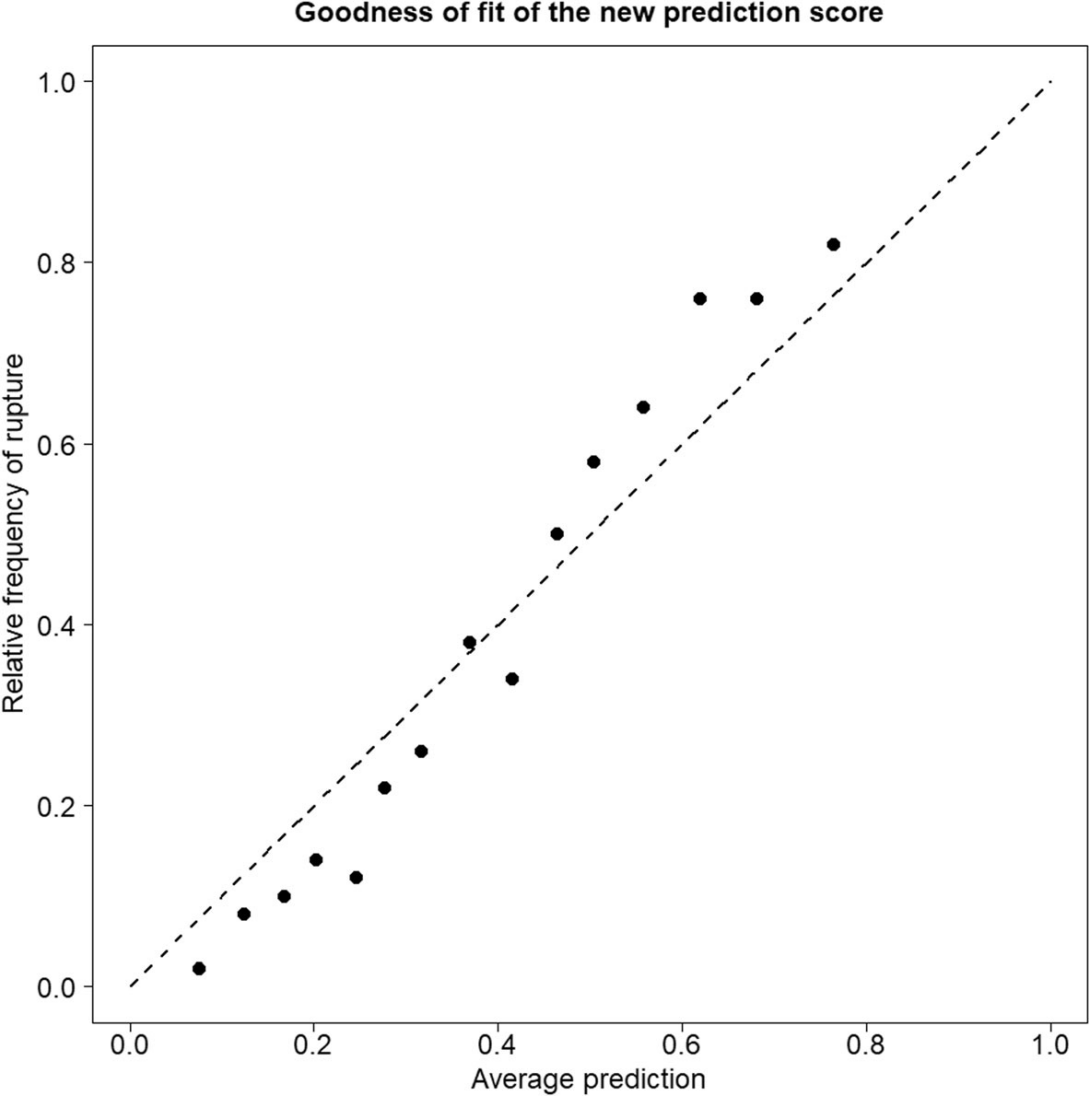
Goodness of fit of the prediction score

In Fig. 3 the goodness of fit of the prediction score was evaluated. The fitted probabilities of the complete data set were ordered and split into 15 intervals, which each consist of 50 aneurysms except the last interval. The total number of aneurysms is not divisible by 50 and therefore the remaining aneurysms were added to the last interval. Overall, the prediction score model is well calibrated, as the estimated probabilities match closely the observed relative frequencies of rupture.

**Fig. 4:**
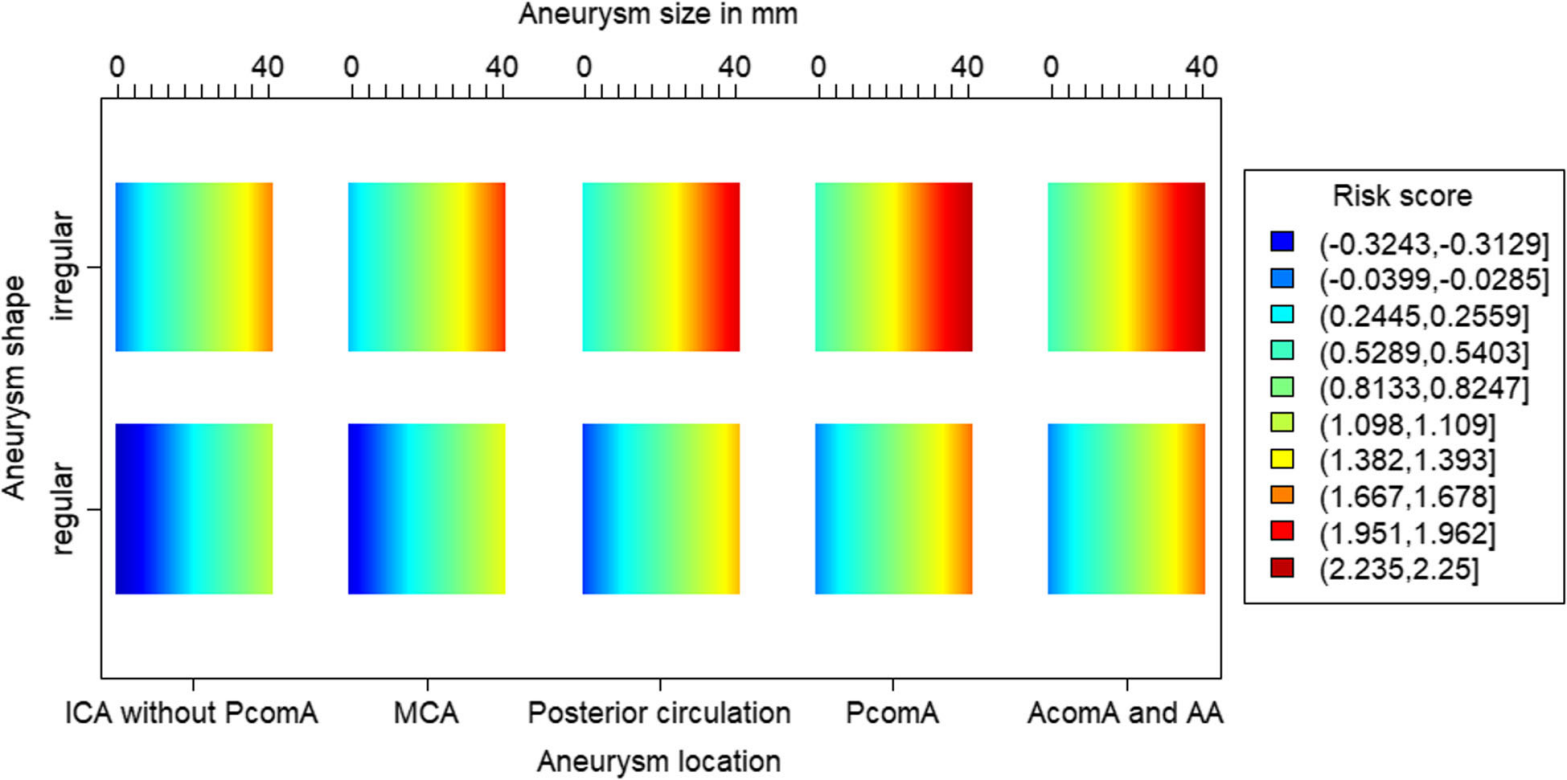
Prediction heatmap for aneurysm rupture. The risk score refers to the minimum and maximum value with colour coding in the right legend. Each bar reflects the distribution of the risk scores from aneurysm size 0 to 40 mm adjusted by aneurysm shape and location AcomA: anterior communicating artery, AA: anterior cerebral artery, ICA: internal carotid artery, MIAs: multiple intracranial aneurysms, MCA: middle cerebral artery, PcomA: posterior communicating artery

Figure 4 shows a heatmap with visual representation of the prediction score parameters.

### Clinical results and validation

After the development of the statistical prediction score, prospective validation was performed utilizing patients with SAH harboring MIAs and presenting with a definitive SAH pattern admitted at the authors’ institution. Cases treated endovascularly, which according to the imaging and hemorrhage pattern the bleeding source remained uncertain, so that the neurovascular team decided one-stage endovascular treatment of all possible bleeding sources, were excluded from the prospective validation. Patients with an obvious bleeding source were also included, in order to gain a larger prospective test sample, since the prediction score is applicable in all cases. Overall, 34 patients were included in the validation of the prediction score. 14 (41%) patients underwent surgical treatment, 16 (47%) patients underwent endovascular treatment, 3 (9%) patients underwent combined surgical and endovascular treatment, and 1 patient (3%) did not underwent any treatment. Overall, 42 of 83 (50%) aneurysms were treated in the acute phase after the ictus. In detail, 19 aneurysms were treated surgically and 23 endovascularly (Table 2).

**Table 2:** prospective patient cohort

Pat. No.	Aneurysm localisation	Aneurysm size (mm)	Aneurysm form	Treatment	Expert opinion before treatment	Score	Surgical Validation	Expert Validation	Positive Score Prediction (YES/NO)
1	MCA left	7	irregular	surgical	MCA	0.4321	YES	YES	**YES**
	AcomA	3	regular	no treatment		0.1281			
2	PcomA	6	regular	surgical	PcomA	0.0216	YES	YES	**YES**
	Basilar tip	2	regular	no treatment		−0.0977			
3	MCA left	7	regular	surgical	MCA left	−0.1066	YES	YES	**YES**
	MCA right	5	regular	no treatment		−0.192			
4	PcomA	6	irregular	endovascular	PcomA	0.7845	NO	YES	**YES**
	A3	3	regular	no treatment		0.1281			
5	AcomA	7	irregular	endovascular	AcomA	0.8376	YES	YES	**YES**
	PcomA	7	regular	combined		0.2885	YES		
	MCA	6	regular	no treatment		−0.1493			
	A1	3	regular	endovascular		0.1281			
	ICA	4	regular	no treatment		−0.4265			
	P1	2	regular	no treatment		−0.0977			
	Basilar tip	3	regular	no treatment		−0.055			
6	Basilar tip	10	regular	endovascular	Basilar tip	0.2439	NO	YES	**YES**
	Basilar side	3	regular	no treatment		−0.055			
7	PcomA	6	irregular	endovascular	PcomA	0.7845	NO	YES	**YES**
	MCA	4	regular	no treatment		−0.2347			
8	AcomA	5	regular	endovascular	AcomA	0.2135	NO	YES	**YES**
	MCA	2	regular	no treatment		−0.3201			
9	AcomA	5	irregular	surgical		0.7522	YES		
	MCA	13	regular	surgical	MCA	0.1496	YES	NO	**YES**
10	AcomA	3	regular	endovascular	AcomA	0.1281	NO	YES	**YES**
	MCA	2	regular	no treatment		−0.3201			
11	MCA left	17	regular	surgical	MCA left	0.3204	YES	YES	**YES**
	MCA right	7	regular	no treatment		−0.1066			
12	Basilar tip	6	irregular	endovascular	Basilar tip	0.6118	NO	YES	**YES**
	PcomA	3	regular	endovascular (staged treatment)		0.1177			
13	AcomA	3	regular	endovascular	AcomA	0.1281	NO	YES	**YES**
	PcomA	3	regular	endovascular		0.1177			
	OpthalmicA	2	regular	no treatment		−0.5119			
	VA	2	regular	no treatment		−0.0977			
14	A3	4	regular	endovascular	A3	0.1708	NO	YES	**YES**
	MCA	3	regular	no treatment		−0.2774			
	MCA	1	regular	no treatment		−0.3628			
15	PcomA right	8	regular	surgical	PcomA	0.3312	YES	YES	**YES**
	PcomA left	4	regular	no treatment		0.1604			
16	MCA left	10	regular	surgical	MCA left	0.0215	YES	YES	**YES**
	MCA right	2	regular	no treatment		−0.3201			
17	PICA left	5	regular	endovascular	PICA	0.0304	NO	YES	**YES**
	SCA left	2	regular	no treatment		−0.0977			
18	VB junction	9	regular	endovascular	VB junction	0.2012	NO	YES	**YES**
	AcomA	4	regular	no treatment		0.1708			
	A2	2	regular	no treatment		0.0854			
19	MCA right	4	regular	no treatment (aSDH right)	MCA right	−0.2347	NO	YES	**YES**
	MCA left	2	regular	no treatment		−0.3201			
20	MCA	8	irregular	surgical	MCA	0.4748	YES	YES	**YES**
	ICA	4	regular	no treatment		−0.4265			
	A3	3	regular	no treatment		0.1281			
21	MCA left	5	regular	surgical	MCA left	−0.192	YES	YES	**YES**
	MCA right	5	regular	no treatment		−0.192			
22	MCA left	9	irregular	surgical	MCA left	0.5175	YES	YES	**YES**
	MCA right	9	regular	surgical (2 stage treatment)		−0.0212	YES	YES	**YES**
23	AcomA	9	irregular	endovascular	AcomA	0.923	NO	YES	**YES**
	ICA	7	regular	no treatment		−0.2984			
24	Basilar tip	6	irregular	endovascular		0.6118	NO	NO	**YES**
	MCA right	10	regular	surgical	MCA	0.0215	YES	NO	**YES**
25	MCA right	8	regular	surgical	MCA right	−0.0639	YES	YES	**YES**
	MCA left	4	regular	no treatment		−0.2347			
	ICA left	4	regular	no treatment		−0.4265			
26	MCA right	7	regular	surgical	MCA right	−0.1066	YES	YES	**YES**
	M2 right	2	regular	surgical		−0.3201	YES		
	MCA links	3	regular	no treatment		−0.2774			
27	PcomA left	7	regular	endovascular	PcomA	0.2885	NO	YES	**YES**
	ICA right	7	regular	endovascular		−0.2984			
28	M2 right	10	regular	surgical	M2 right	0.0215	YES	YES	**YES**
	ICA left	6	regular	no treatment		−0.3411			
	M2 left	3	regular	no treatment		−0.2774			
29	ICA	9	regular	endovascular	ICA	−0.213	NO	YES	**YES**
	MCA	3	regular	no treatment		−0.2774			
30	PcomA	10	regular	endovascular	PcomA	0.4166	NO	YES	**YES**
	AcomA	4	regular	no treatment		0.1708			
31	AcomA	11	regular	endovascular	AcomA	0.4697	NO	YES	**YES**
	PcomA	5	regular	no treatment		0.2031			
32	ACA (A3)	9	regular	endovascular	ACA (A3)	0.3843	NO	YES	**YES**
	MCA left	3	regular	no treatment		−0.2774			
	ICA left	2	regular	no treatment		−0.5119			
	SCA right	6	regular	no treatment		0.0731			
33	AcomA	5	regular	endovascular	AcomA	0.2135	YES	YES	**YES**
	M3 right	3	regular	surgical		−0.2774	YES		
34	PcomA right	9	regular	surgical	PcomA	0.3739	YES	YES	**YES**
	ICA left	6	regular	no treatment		−0.3411			

*A1* segment 1 of anterior cerebral artery, *A2* segment 2 of anterior cerebral artery, *A3* segment 3 of anterior cerebral artery, *AA* anterior cerebral artery, *AcomA* anterior communicating artery, *aSDH* acute subdural hematoma, *ICA* internal carotid artery, *M2* segment 2 of middle cerebral artery, *M3* segment 3 of middle cerebral artery, *MCA* middle cerebral artery, *OphtalmicA* ophthalmic artery, *P1* segment 1 of posterior cerebral artery, *PcomA* posterior communicating artery, *PICA* posterior inferior cerebellar artery, *SCA* superior cerebellar artery, *VB* vertebrobasilar

### Challenging cases

The prediction score identified the ruptured aneurysm in all 34 prospectively analyzed cases correctly, while the neurovascular team was correct in 32 cases. In two surgically treated patients (Pat. No. 9 and 24), the assumption of the neurovascular team was incorrect, while the prediction score was correct. In the first case, both aneurysms were treated surgically in the same session (MCA and AcomA), where the assumed ruptured aneurysm (MCA) was inspected and identified as unruptured, while the other aneurysm (AcomA) was identified as the bleeding source (illustrative case). In the second case, the bleeding source was uncertain for the neurovascular team. Therefore, the basilar tip artery aneurysm was treated endovascularly at first (assumed as the most likely bleeding source), and the MCA aneurysm was treated surgically secondarily. During surgical inspection, the MCA aneurysm was identified as the bleeding source.

In three other cases, patients were treated in the acute phase on their MIAs surgically. During surgical inspection the presumably ruptured aneurysm according to the prediction score, was identified as the bleeding source (Pat. No. 5, 26 and 33).

Patient 5 harbored seven aneurysms. The neurovascular team suspected the AcomA aneurysm as the bleeding source. Because of the proximity of a smaller A1 aneurysm, a clear distinction of the bleeding source was however not possible. On the other hand, the PcomA aneurysm also could not be ruled out as probable bleeding source. After coiling of the AcomA and A1 aneurysms and incomplete coiling of the PcomA aneurysm, surgical treatment of the PcomA aneurysm was decided. During surgical inspection, the AcomA aneurysm was identified as the bleeding source.

Patient 26 harbored two MCA aneurysms on the right side in proximity. Even if the size of one of them was small, surgical treatment was performed, because rupture of the smaller aneurysm could not be ruled out as bleeding source. During surgical treatment the larger one was identified as the bleeding source.

Patient 33 harbored two aneurysms (AcomA and MCA). After endovascular treatment of the AcomA aneurysm, the MCA aneurysm was treated surgically in the acute phase and did not show any sign of rupture.

Therefore, the prediction score assigned the ruptured aneurysm correctly in overall 17 patients in whom at least one aneurysm was treated surgically.

#### Endovascularly treated patients

In all 19 patients treated endovascularly, and in the one patient without treatment, the prediction score correlated with the assumption of the neurovascular team, and the distinct bleeding pattern in the CT-scans, indicating the ruptured aneurysm.

None of the patients treated surgically or endovascularly, suffered from rehemorrhage throughout the follow-up of 12 months.

### Illustrative case

**Fig. 5:**
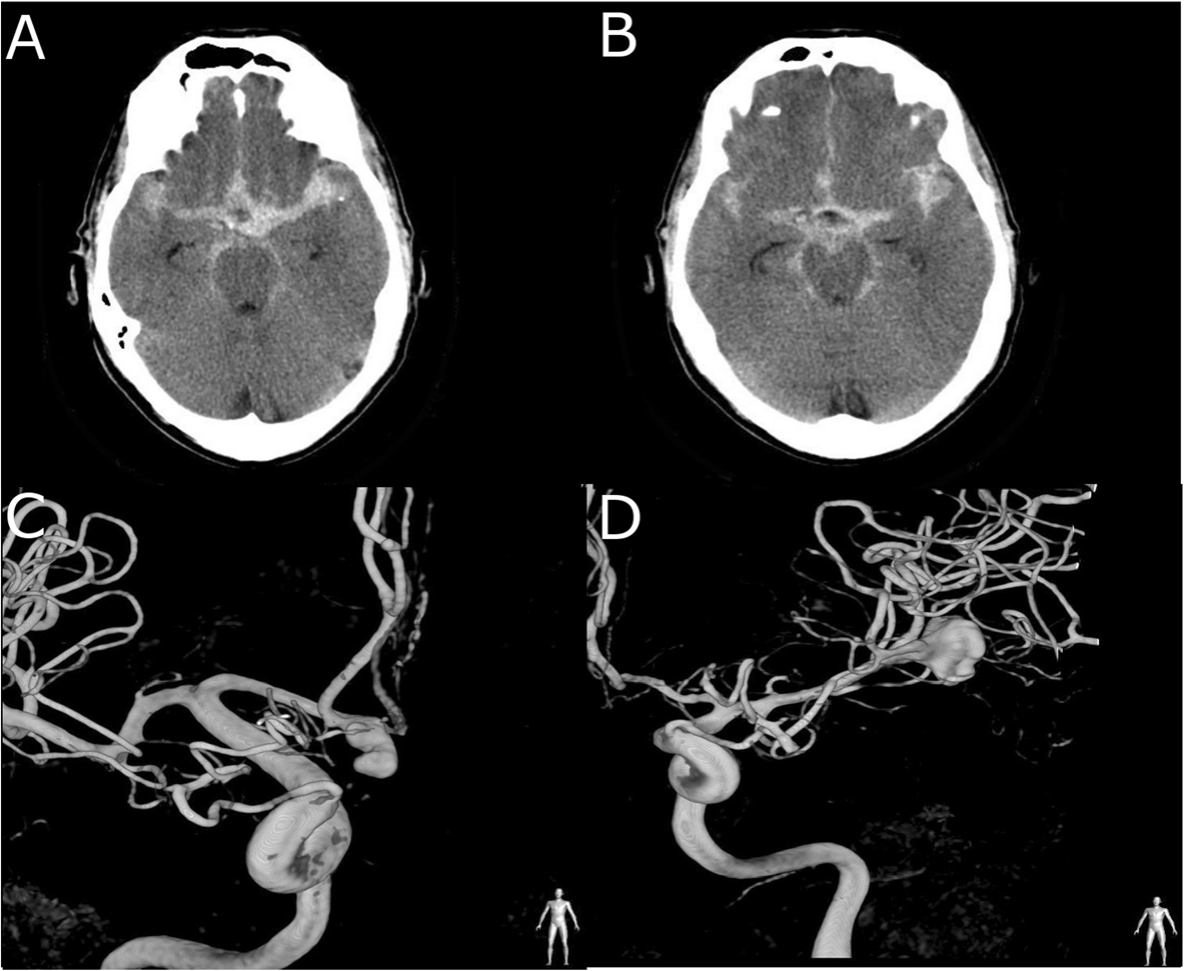
Illustrative case. A and B: axial native CT scan showing SAH at admission, C: DSA showing the AcomA aneurysm at admission, D: DSA showing the left MCA aneurysm at admission

The patient was admitted with acute onset of headache at the author’s institution (Fig. 5). The patient harbored two aneurysms; one regular shaped 13 mm aneurysm at the bifurcation of the left MCA, and one irregular shaped 5 mm aneurysm at the AcomA. The neurovascular team assumed the rupture of the aneurysm located at the bifurcation of the MCA, because of the pattern of the hemorrhage in the left sylvian fissure and the size of the aneurysm. After interdisciplinary decision, the patient was treated surgically. The intraoperative inspection revealed that the aneurysm located at the AcomA was ruptured, and the MCA aneurysm was not. Both aneurysms were successfully treated by clipping.

### Score calculation

1) AcomA: 5 (size) × 0.0427 + 0 (location) + 0.5387 (shape) = 0.7522.

2) MCA: 13 (size) × 0.0427–0.4055 (location) + 0 (shape) = 0.1496.

Therefore, the prediction score (being larger for the AcomA aneurysm) predicted correctly the ruptured aneurysm.

## Discussion

Definite treatment of a ruptured aneurysm is of highest priority in patients with aneurysmal SAH [7, 8]. In patients with SAH harboring MIAs, identifying the ruptured aneurysm may be challenging. Identification of the ruptured aneurysm in case of MIAs is ascertained from the pattern of hemorrhage and other parameters, such as shape, location and size of the aneurysm [9]. Misidentification of the ruptured aneurysm and treatment of a falsely assumed ruptured aneurysm could have major impact on the outcome of the patient, since rebleeding and severe brain injury secondary to rebleeding can occur and are known predictors of poor outcome [10, 11]. On the other hand, treatment of additional unruptured aneurysms in patients with MIAs in the acute phase of the SAH is discussed controversially due to the risk of additional complications, and is therefore usually performed secondarily [12]. Depending on the size and location of the remaining unruptured aneurysms and the clinical condition of the patients, secondary treatment is applied in interdisciplinary consensus.

### Hemorrhage pattern

Orning et al. [9] showed that in cases of definite hemorrhage pattern, where the hemorrhage is clearly lateralizing or otherwise confining to an aneurysm, identification of the ruptured aneurysm is highly accurate. However, in the same study there was a high inaccuracy in cases of nondefinite hemorrhage pattern. Uncertainty arises in cases with a diffuse and symmetric hemorrhage pattern, or a localized pattern, but with multiple aneurysms in that particular area are present in the same patient.

Because this prediction score was developed for the uncommon but usually troublesome cases, where the hemorrhage pattern does not provide any further clues for the identification of the bleeding source, CT findings were deliberately not included in the score. Given the fact that in all cases the CT findings would not provide further information, the value of this parameter would be constant for each aneurysm without any influence on the score.

### Aneurysm size and location

According to previous studies, size and location are postulated as independent risk factors for aneurysm rupture [13–18]. According to the ISUIA trial, aneurysms located at the posterior circulation including the PcomA, have a higher possibility of rupture [15]. However, Juvela et al. postulated that aneurysms located at the ACA were at significant risk to rupture [1]. In patients with MIAs, aneurysms located at the AcomA had the highest probability to rupture according to Nehls et al. [3].

According to the findings of the present study, the site with the highest probability of a ruptured aneurysm in patients harboring MIAs was the AA including the AcomA. Furthermore, aneurysm size was also identified to be a risk factor for aneurysm rupture.

Backes et al. [19] reported that the ruptured aneurysm in patients with MIAs was not the largest aneurysm in 29% of the patients. Therefore, the use of aneurysm size or aneurysm location alone seems not to predict the ruptured aneurysm adequately.

### Aneurysm shape

Irregular aneurysm shape is considered to be associated with aneurysm enlargement which is a surrogate parameter for aneurysm rupture [20, 21]. Backes et al. [19] described this parameter as a factor for aneurysm rupture independent of aneurysm size, location and patient characteristics in MIAs. Maslehaty et al. [22] investigated the anatomical factors in cases of MCA mirror aneurysms and showed that size and shape were predictive for rupture in their series. Nehls et al. found, that irregularity of the aneurysm morphology was more indicative for the ruptured aneurysm than size [3]. In the present study, irregular shaped aneurysms were also found to rupture more likely compared to regularly shaped aneurysms.

### Clinical setting and use of the prediction score

Aim of the development of the scoring system was to create a simple tool, to identify the ruptured aneurysm in patients with MIAs and unclear bleeding pattern in cases of SAH. We only used readily available data, that was accessible using CT-A or DSA. Therefore, the described prediction score can easily be assessed and used in the clinical setting. According to our findings and experience using the prediction score, for example in the illustrative case, it can provide additional information and improve the treatment decision. The score is developed to be used in cases with diffuse SAH, without distinct bleeding pattern pointing out the bleeding source. In the prospective validation dataset, five of the cases (15%) were challenging in respect of defining the bleeding source. In all these challenging cases, for which the prediction score was initially developed, the bleeding source was identified correctly. In contrast, the neurovascular team predicted the true bleeding source in three of the five patients correctly. Given the uncertainty in some challenging cases, treatment of all possible bleedings sources was performed. The true value of the prediction score might be in troublesome cases, where treatment of multiple aneurysms might not be easily feasible due to aneurysm or patient specific characteristics. The prediction model can provide additional information for the decision-making process.

### Limitations

The score was derived from a retrospective dataset of the authors institution and was then prospectively validated at the same center. Furthermore, the prospective validated score was conducted with a relatively small number of patients, since patients harboring MIAs with SAH represent just a small fraction of all SAH patients and the troublesome cases with non-definite bleeding pattern are infrequent. The prediction score was additionally validated with endovascularly treated aneurysms, in order to gain a greater number of patients. In all patients treated endovascularly, the bleeding pattern pointed unambiguously the ruptured aneurysm. However, if only the surgically treated aneurysms were to be included in the validation, the correctness of the prediction score would remain by 100%.

The score is not intended to replace the expert’s decision. It is supposed to be a cornerstone for further multicenter prospective studies for its validation, where afterwards it can be used to support neurovascular team’s decisions. Given the fact that the challenging cases needed for prospective validation are overall rare, multicenter independent data is necessary.

## Conclusions

This simple prediction score might provide support for neurovascular teams for treatment decision in SAH patients harboring multiple intracranial aneurysms and no definite hemorrhage pattern in order to identify the ruptured aneurysm. However, larger cohorts for prospective evaluation are warranted.

### Disclosures

The authors report no conflict of interest concerning the materials or methods used in this study or the findings specified in this paper.

## Data Availability

The datasets used and/or analyzed during the current study are available from the corresponding author on reasonable request.

## Abbreviations

A1: Segment 1 of anterior cerebral artery
A2: Segment 2 of anterior cerebral artery
A3: Segment 3 of anterior cerebral artery
AA: Anterior cerebral artery
AcomA: Anterior communicating artery
AHT: Hypertension
aSDH: Acute subdural hematoma
CT: Computed tomography
CT-A: CT – angiography
DSA: Digital subtraction angiography
ICA: Internal carotid artery
ICH: Intracerebral hemorrhage
M2: Segment 2 of middle cerebral artery
M3: Segment 3 of middle cerebral artery
MCA: Middle cerebral artery
MIAs: Multiple intracranial aneurysms
OphtalmicA: Ophthalmic artery
P1: Segment 1 of posterior cerebral artery
PcomA: Posterior communicating artery
PICA: Posterior inferior cerebellar artery
SAH: Subarachnoid hemorrhage
SCA: Superior cerebellar artery
VB: Vertebrobasilar
